# The Identification and Evaluation of Interleukin-7 as a Myokine Biomarker for Peripheral Artery Disease Prognosis

**DOI:** 10.3390/jcm13123583

**Published:** 2024-06-19

**Authors:** Ben Li, Farah Shaikh, Abdelrahman Zamzam, Muzammil H. Syed, Rawand Abdin, Mohammad Qadura

**Affiliations:** 1 Department of Surgery, University of Toronto, Toronto, ON M5S 1A1, Canada; benx.li@mail.utoronto.ca; 2 Division of Vascular Surgery, St. Michael’s Hospital, Unity Health Toronto, University of Toronto, 30 Bond Street, Suite 7-076, Toronto, ON M5B 1W8, Canada; farah.shaikh@unityhealth.to (F.S.); abdelrahman.zamzam@gmail.com (A.Z.); muzammil.syed@mail.utoronto.ca (M.H.S.); 3 Institute of Medical Science, University of Toronto, Toronto, ON M5S 1A1, Canada; 4 Temerty Centre for Artificial Intelligence Research and Education in Medicine, University of Toronto, Toronto, ON M5S 1A1, Canada; 5 Department of Medicine, McMaster University, Hamilton, ON L8S 4L8, Canada; rawand.abdin@medportal.ca; 6 Li Ka Shing Knowledge Institute, St. Michael’s Hospital, Unity Health Toronto, University of Toronto, Toronto, ON M5B 1W8, Canada

**Keywords:** myokines, interleukin-7, predictive model, prognosis, peripheral artery disease

## Abstract

**Background/Objectives:** Myokines have been demonstrated to be associated with cardiovascular diseases; however, they have not been studied as biomarkers for peripheral artery disease (PAD). We identified interleukin-7 (IL-7) as a prognostic biomarker for PAD from a panel of myokines and developed predictive models for 2-year major adverse limb events (MALEs) using clinical features and plasma IL-7 levels. **Methods:** A prognostic study was conducted with a cohort of 476 patients (312 with PAD and 164 without PAD) that were recruited prospectively. Their plasma concentrations of five circulating myokines were measured at recruitment, and the patients were followed for two years. The outcome of interest was two-year MALEs (composite of major amputation, vascular intervention, or acute limb ischemia). Cox proportional hazards analysis was performed to identify IL-7 as the only myokine that was associated with 2-year MALEs. The data were randomly divided into training (70%) and test sets (30%). A random forest model was trained using clinical characteristics (demographics, comorbidities, and medications) and plasma IL-7 levels with 10-fold cross-validation. The primary model evaluation metric was the F1 score. The prognostic model was used to classify patients into low vs. high risk of developing adverse limb events based on the Youden Index. Freedom from MALEs over 2 years was compared between the risk-stratified groups using Cox proportional hazards analysis. **Results:** Two-year MALEs occurred in 28 (9%) of patients with PAD. IL-7 was the only myokine that was statistically significantly correlated with two-year MALE (HR 1.56 [95% CI 1.12–1.88], *p* = 0.007). For the prognosis of 2-year MALEs, our model achieved an F1 score of 0.829 using plasma IL-7 levels in combination with clinical features. Patients classified as high-risk by the predictive model were significantly more likely to develop MALEs over a 2-year period (HR 1.66 [95% CI 1.22–1.98], *p* = 0.006). **Conclusions:** From a panel of myokines, IL-7 was identified as a prognostic biomarker for PAD. Using a combination of clinical characteristics and plasma IL-7 levels, we propose an accurate predictive model for 2-year MALEs in patients with PAD. Our model may support PAD risk stratification, guiding clinical decisions on additional vascular evaluation, specialist referrals, and medical/surgical management, thereby improving outcomes.

## 1. Introduction

Peripheral artery disease (PAD) is characterized by atherosclerosis in the arteries of the lower extremities, leading to reduced blood flow and limb ischemia [1]. This condition, affecting more than 200 million individuals worldwide, presents with symptoms such as claudication, rest pain, and tissue damage [2]. Despite its strong correlation with limb loss and mortality, PAD often receives inadequate treatment [3]. A potential approach to addressing this issue involves the identification of biomarkers to aid in the prognosis of PAD patients [4,5]. 

Myokines refer to cytokines and other peptides produced, expressed, and released by muscle fibers, exerting autocrine, paracrine, or endocrine effects [6]. Given that PAD is characterized by muscle ischemia, myokines may act as biomarkers for PAD development and progression [6]. Our group has previously identified several biomarkers for PAD, including fatty acid binding proteins [5,7,8,9,10], inflammatory proteins [11], and Cystatin C [12]; however, the investigation of myokines as PAD biomarkers has been limited. Myokines, including fibroblast growth factor-23 (FGF-23) [13], tumour necrosis factor-related apoptosis-inducing ligand receptor 2 (TRAIL-R2) [14], interleukin-7 (IL-7) [15], and monocyte chemoattractant protein-1 (MCP-1) [16], among others, have been implicated in cardiovascular diseases. In fact, many myokines have been demonstrated to be associated with cardiovascular diseases such as cerebrovascular disease (CVD), coronary artery disease (CAD), and PAD [17,18,19,20,21]. The selection of these five specific myokines for analysis in this study stems from their extensive investigation and robust association with cardiovascular diseases, suggesting potential relevance to PAD [17,18,19,20,21]. While past research has indicated correlations between these proteins and cardiovascular conditions, few studies have specifically delved into their prognostic implications for PAD [17,18,19,20,21]. Given the multifactorial nature of PAD and its chronic development involving diverse metabolic pathways, our hypothesis posits that an integrated model consisting of clinical features and biomarker data can enhance prognostic accuracy compared to the analysis of individual proteins or clinical features alone [22]. Combining myokine biomarker data with demographic and clinical characteristics associated with PAD outcomes holds potential for developing highly accurate predictive algorithms for adverse limb events linked to PAD [23,24,25]. The objective of this study is to identify PAD-specific prognostic biomarkers from a panel of myokines and integrate clinical and myokine biomarker data to build prognostic models for PAD that can guide clinical decision-making. 

## 2. Materials and Methods

### 2.1. Ethics

This study received approval from the research ethics board at Unity Health Toronto, the University of Toronto, Canada, on 8 February 2017 (REB # 16-365). Before participating, all individuals provided informed consent, and all the procedures strictly followed the principles outlined in the Declaration of Helsinki [26]. 

### 2.2. Design

This was a prognostic study, with the findings reported in alignment with the Transparent Reporting of a Multivariable Prediction Model for Individual Prognosis or Diagnosis + Artificial Intelligence (TRIPOD+AI) statement [27]. Specifically, PAD-specific prognostic biomarkers were identified from a pool of circulating myokines, and these biomarkers were used in combination with relevant clinical features to develop a predictive model for adverse limb events in patients with PAD.

### 2.3. Patient Recruitment

This study involved the prospective recruitment of patients, both with and without PAD, who sought care at ambulatory clinics within our institution from September 2020 to February 2022. PAD was identified by an Ankle–Brachial Index (ABI) of less than 0.9 or a Toe–Brachial Index (TBI) of less than 0.67 and a hemodynamically significant stenosis > 50% in a lower extremity artery on duplex ultrasound, coupled with absent or diminished pedal pulses [28]. Conversely, non-PAD was defined by an ABI of 0.9 or higher, a TBI of 0.67 or higher, the absence of a hemodynamically significant stenosis in the lower extremity arteries on duplex ultrasound, and normal pedal pulses [28]. The exclusion criteria encompassed patients with elevated troponin levels, acute coronary syndrome, or acute limb ischemia within the preceding three months.

### 2.4. Baseline Characteristics

The baseline characteristics documented in this study encompassed age, gender, hypertension (diastolic blood pressure ≥ 80 mmHg, systolic blood pressure ≥ 130 mmHg, or receiving blood-pressure-lowering therapy [29,30]), dyslipidemia (triglyceride > 1.7 mmol/L, total cholesterol > 5.2 mmol/L, or receiving lipid-lowering therapy [29,30]), diabetes (hemoglobin A1c ≥ 6.5% or receiving an antidiabetic medication [29,30]), current or past smoking status, the presence of congestive heart failure (CHF), coronary artery disease (CAD), history of stroke, and the use of cardiovascular risk reduction medications, including statins, acetylsalicylic acid (ASA), angiotensin-converting enzyme inhibitors (ACE-Is) or angiotensin II receptor blockers (ARBs), calcium channel blockers, beta-blockers, hydrochlorothiazide or furosemide, oral antihyperglycemic agents, and insulin. These definitions for cardiovascular risk factors and medications were based on guidelines from the American College of Cardiology [29,30].

### 2.5. Quantification of Plasma Myokine Levels

Blood samples were obtained from the patients, and their plasma concentrations of 5 circulating myokines were assessed in duplicate using the LUMINEX assay (Bio-Techne, Minneapolis, MN, USA) [31]. The selection of the following proteins—FGF-23, TRAIL-R2, IL-7, MCP-1, and leukemia inhibitory factor (LIF)—was based on their involvement in various metabolic processes linked to atherosclerosis and their significant associations with cardiovascular diseases. LIF was selected as a potential PAD biomarker because it has been found to be implicated in cardiovascular diseases secondary to its role in promoting cardiac hypertrophy [32], involvement in the pathophysiology of heart failure [33], and contribution to neovascularization [34]. Given that these biological processes are highly relevant to PAD development and progression, LIF was investigated as a potential PAD biomarker. The analysis of multiple myokines seeks to identify new biomarkers for PAD. Before the sample analysis, Fluidics Verification and Calibration bead kits from Luminex Corp [35] were employed to calibrate the MagPix analyzer (Luminex Corp.; Austin, Texas) [36]. To minimize inter-assay variability, all the sample analyses were conducted on the same day. Both intra-assay and inter-assay coefficients of variability were maintained at <10%. At least 50 beads for each myokine were acquired and analyzed using Luminex xPonent software version 4.3 [37].

### 2.6. Follow-Up and Outcomes

Outpatient clinic visits were scheduled 1 year and 2 years post-baseline assessment. The primary outcome of interest was the occurrence of major adverse limb events (MALEs) over the 2-year period. MALEs were defined as the necessity for vascular intervention (either open or endovascular lower extremity revascularization), major lower extremity amputation above the ankle, or acute limb ischemia (sudden decrease in limb perfusion [<14 days] caused by arterial thrombosis or embolism). The initial analysis revealed that all adverse limb events occurred in patients diagnosed with PAD. Consequently, prognostic models for predicting MALEs were developed exclusively for the PAD cohort.

### 2.7. Model Development and Evaluation

The selected predictive model for this study was the random forest, an ensemble learning technique that operates by utilizing multiple decision trees [38]. Decision trees partition populations into branch-like segments, and by leveraging various covariates, they construct prediction algorithms for a target outcome [39]. Due to its non-parametric nature, random forest can effectively handle large and intricate datasets [39]. This algorithm was chosen because of its widespread use in the literature and its demonstrated high performance in predicting health outcomes [40,41,42]. 

The dataset was randomly divided into 70% training and 30% test sets. The random forest algorithm underwent training using 10-fold cross-validation to predict both primary and secondary outcomes. The input features included clinical characteristics (age, sex, hypertension, dyslipidemia, diabetes, past/current smoking, CHF, CAD, previous stroke, ASA, statins, ACE-Is or ARBs, beta-blockers, calcium channel blockers, hydrochlorothiazide or furosemide, oral antihyperglycemic agents, and insulin) and plasma IL-7 levels. IL-7 was chosen as the myokine biomarker of interest as it was the only myokine that was significantly associated with PAD-related adverse events. Once trained, the models were evaluated on unseen test set data. The prognostic model was assessed only on patients with PAD given that all the MALE outcomes presented in patients with PAD.

### 2.8. Statistical Analysis

The demographic and clinical characteristics of our cohort were summarized using means and standard deviations (SDs) for continuous variables or numbers and proportions for categorical variables. Baseline differences between groups were assessed using independent t-tests for continuous variables and chi-square tests for categorical variables. The myokine levels were compared between patients with and without PAD using independent t-tests when the data were normally distributed or Mann–Whitney U tests when the data were non-normally distributed. The event rates at 2 years were compared between PAD and non-PAD patients using chi-square tests. Associations between individual myokines and 2-year MALEs were determined using Cox proportional hazards analysis with adjustment for age, sex, hypertension, dyslipidemia, diabetes, past/current smoking, CHF, CAD, previous stroke, ASA, statins, ACE-Is or ARBs, beta blockers, calcium channel blockers, hydrochlorothiazide or furosemide, oral antihyperglycemic agents, and insulin. Myokines that were associated with 2-year MALEs were used to build the predictive model in combination with clinical features. The predictive ability of the model was assessed for the prognosis of 2-year MALEs in the PAD cohort. The primary metric utilized to assess the model performance was the F1 score, which quantifies the harmonic mean of precision and recall values in predicting adverse limb events based on ST2 levels [43]. The F1 score is computed using the formula 2 × ([precision × recall]/[precision + recall]) [43]. It ranges between 0 and 1, with 1 indicating the maximum precision and recall, while 0 indicates no precision and/or recall [43]. Using the prognostic model, patients were stratified into being at low or high risk of developing 2-year MALEs using the Youden Index, which optimizes the performance (sensitivity and specificity) of the prediction model through receiver operating characteristic curve analysis [44]. The analysis of freedom from MALEs over 2 years in low- vs. high-risk patients was conducted using Kaplan–Meier curves. These curves were then compared utilizing Cox proportional hazards analysis, with adjustment for the baseline characteristics (sex, age, hypertension, dyslipidemia, diabetes, past/current smoking, CHF, CAD, previous stroke, ASA, statins, ACE-Is or ARBs, beta blockers, calcium channel blockers, hydrochlorothiazide or furosemide, oral antihyperglycemic agents, and insulin). The purpose of this stratified analysis is to understand the potential clinical significance of the risk predictions made by the prognostic model. Specifically, it helps clinicians understand how a low- vs. high-risk patient’s trajectory over a 2-year period differs in terms of MALE risk. Significance was established at a two-tailed *p*-value < 0.05. All the analyses were performed using SPSS software version 23 [45].

## 3. Results

### 3.1. Patients

Overall, 476 patients were enrolled in the study, with 312 diagnosed with PAD and 164 without PAD. The patients with PAD tended to be older (mean age 71 [SD 10] vs. 65 [SD 12] years, *p* < 0.001) and had a higher prevalence of dyslipidemia (84% vs. 61%, *p* < 0.001), hypertension (82% vs. 59%, *p* < 0.001), CAD (38% vs. 21%, *p* < 0.001), diabetes (42% vs. 21%, *p* < 0.001), and a history of stroke (16% vs. 8%, *p* = 0.011). Additionally, they were more likely to be past or current smokers (80% vs. 64%, *p* = 0.002). Furthermore, they had a higher rate of taking risk reduction medications, including statins (73% vs. 57%, *p* < 0.001), ASA (80% vs. 60%, *p* < 0.001), ACE-Is/ARBs (66% vs. 45%, *p* = 0.001), and beta blockers (41% vs. 30%, *p* = 0.001) (Table 1).

### 3.2. Plasma Concentrations of Myokines

Of the five myokines tested, four were significantly elevated in patients with PAD compared to those without PAD based on their median [IQR] plasma concentrations: FGF-23 (15.33 [IQR 10.43–23.67] vs. 12.19 [IQR 9.41–17.39] pg/ml, *p* < 0.001), TRAIL-R2 (32.76 [IQR 24.37–45.84] vs. 25.65 [IQR 19.40–35.12] pg/ml, *p* < 0.001), IL-7 (3.78 [IQR 3.14–5.05] vs. 3.41 [IQR 2.88–4.21] pg/ml, *p* = 0.001), and MCP-1 (98.66 [IQR 69.15–124.90] vs. 88.27 [IQR 67.44–115.88], *p* = 0.031) (Table 2). There were no statistically significant differences in the plasma myokine concentrations between patients with and without CAD, demonstrating the specificity of the myokine biomarkers for PAD (Table 3). 

### 3.3. Adverse Limb Events

All adverse limb events occurred in PAD patients over a follow-up period of 2 years: MALEs (*n* = 28, 9%), vascular intervention (*n* = 19, 6%), major amputation (*n* = 17, 5%), and worsening PAD status (*n* = 56, 18%). No patients developed acute limb ischemia (Table 4). When the proteins were analyzed individually, there were significant associations between IL-7 and 2-year MALEs (HR 1.56 [95% CI 1.12–1.88], *p* = 0.007), major amputation (HR 1.02 [95% CI 1.01–1.88], *p* = 0.042), and vascular intervention (HR 1.10 [95% CI 1.05–2.98], *p* = 0.019). There were no statistically significant associations between TRAIL-R2, MCP-1, or FGF-23 and 2-year MALEs, vascular intervention, or major amputation. Therefore, IL-7 was chosen as the PAD-specific prognostic biomarker and used in further analyses (Table 5). Although FGF-23 and TRAIL-R2 achieved lower *p*-values (< 0.001) in the PAD vs. non-PAD comparison relative to IL-7 (*p* = 0.001), there was no statistically significant association between FGF-23 and TRAIL-R2 and 2-year MALEs in the HR analysis (*p* values of 0.484 and 0.635, respectively), while there was a statistically significant association between IL-7 and 2-year MALEs (*p* = 0.007). Since the goal was to identify a prognostic biomarker that could predict 2-year MALEs in patients with PAD, IL-7 was deemed to be a more suitable candidate as a PAD prognostic biomarker given its strong association with 2-year MALEs (HR 1.56 [95% CI 1.12–1.88]). 

### 3.4. Model Performance

**Figure 1:**
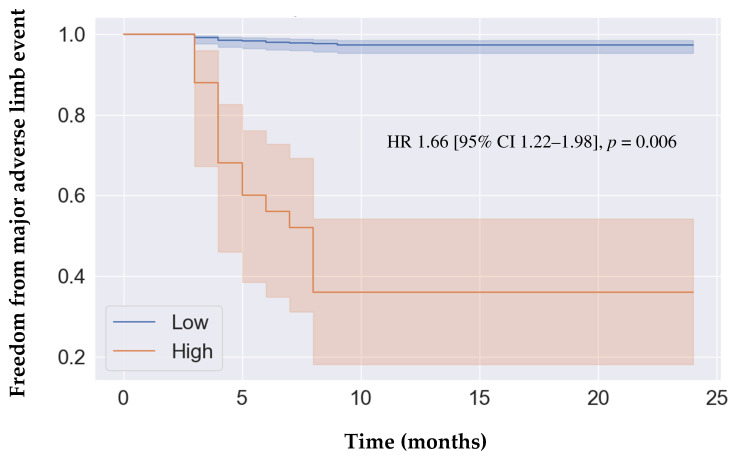
Kaplan–Meier analysis of freedom from major adverse limb events in patients predicted to be at low vs. high risk by random forest model. The threshold used to classify patients into low- vs. high-risk was 0.60 based on the Youden Index, which optimizes model performance (sensitivity and specificity) using receiver operating characteristic curve analysis. Cox proportional hazards analysis adjusted for sex, age, dyslipidemia, hypertension, past/current smoking, diabetes, coronary artery disease, congestive heart failure, previous stroke, statin, acetylsalicylic acid, angiotensin converting enzyme inhibitors or angiotensin II receptor blockers, calcium channel blockers, beta blockers, calcium channel blockers, hydrochlorothiazide or furosemide, oral antihyperglycemic agents, and insulin. Abbreviations: HR (hazard ratio), CI (confidence interval).

IL-7 was selected as the biomarker of interest to build the prognostic model because it was the only myokine that demonstrated a statistically significant association with 2-year MALEs with an HR of 1.56 [95% CI 1.12–1.88], *p* = 0.007. The purpose of correlating IL-7 with 2-year MALEs was to demonstrate the association of this biomarker with adverse limb events in patients with PAD. The reason for using HRs to demonstrate the association between IL-7 and 2-year MALEs was to control for confounding variables to assess the independent relationship between IL-7 and 2-year MALEs. Using a combination of clinical features and plasma IL-7 levels, the random forest model achieved an F1 score of 0.829 for predicting 2-year MALEs, indicating excellent precision and recall. Calculation of Youden’s Index demonstrated that the best cut-off threshold for the prediction of 2-year MALEs using the model combining clinical features and plasma IL-7 levels was 0.60. This threshold score was used to stratify our cohort into patients predicted to be at high vs. low risk of adverse limb events. Over a 2-year period, patients predicted to be at high risk had a lower freedom from MALEs compared to patients predicted to be at low risk (HR 1.66 [95% CI 1.22–1.98], *p* = 0.006) (Figure 1).

## 4. Discussion

### 4.1. Summary of Findings

In this study, we identified IL-7 as a prognostic biomarker for PAD and developed a robust model using a combination of clinical characteristics and plasma IL-7 levels that accurately predicts PAD prognosis. Several key findings emerged from our analysis. First, from the five circulating myokines analyzed, we found that IL-7 was the only myokine that was significantly associated with PAD-related adverse limb events, including 2-year MALEs, vascular intervention, and major amputation. Second, we developed a predictive model using clinical features and plasma IL-7 levels that achieved excellent performance for predicting 2-year MALEs in patients with PAD. Given the significance of IL-7 in PAD prognosis, further basic science and translational research is warranted to elucidate the biological relationships between these proteins and PAD development/progression, with the goal of informing targeted therapeutics. Third, we used our prognostic model including clinical features and plasma IL-7 levels to classify patients into being at low vs. high risk of developing adverse events. Using Kaplan–Meier analysis, we demonstrated that patients classified as high-risk by our model were more likely to develop MALEs over a 2-year period compared to low-risk patients. This demonstrates the clinical relevance of our IL-7-based model in helping clinicians understand the future trajectory of their PAD patients in terms of the risk of adverse limb events. 

### 4.2. Comparison to Existing Literature

Ross et al. (2019) developed a model to predict Major Adverse Cardiac and Cerebrovascular Events (MACCE) in PAD patients using data extracted from electronic health records [46]. In their study, the authors developed models based on retrospectively collected information, including International Classification of Diseases (ICD)-9 codes, Common Procedural Terminology (CPT) codes, prescriptions, and other clinical data [46]. However, a limitation of their study was the lack of biomarker-based data as input features, despite the demonstrated impact of such information on PAD prognosis, as evidenced by our study and others [47,48,49,50]. Our study addressed this limitation by considering myokine biomarker data (plasma IL-7 levels) as an input feature in our ML models. We achieved excellent performance metrics for PAD prognosis, with an F1 score of 0.829 for predicting 2-year MALEs. Therefore, we demonstrate the value of building ML models using biomarker information, which can improve predictive performance compared to clinical characteristics alone.

### 4.3. Explanation of Findings

There are several potential explanations for our findings. IL-7 was found to be an important predictor of PAD prognosis, as it may be involved in various mechanistic pathways important for cardiovascular disease development and progression [15]. IL-7 is a 25 kDa soluble globular protein produced by stromal cells in the bone marrow, thymus, and other epithelial cells [51]. The IL-7 receptor is a heterodimeric complex consisting of the α-chain CD127 and the common cytokine receptor γ-chain, shared with receptors for several other interleukins [51]. Hence, IL-7 exerts multiple biological effects and impacts various cell types by binding to its receptor [51]. Insufficiencies in IL-7 can result in significantly compromised immune cell development [51]. Ultimately, IL-7 serves as a crucial factor in the development and preservation of the immune system [51]. In relation to cardiovascular diseases, IL-7 acts as a regulator of T cell homeostasis with involvement in inflammatory processes and has been demonstrated to drive atherogenesis and promote plaque instability [15]. CD8 T cells that express the memory antigen CD95 have demonstrated pro-atherogenic effects and are associated with cardiovascular disease development in humans [48]. Yan et al. (2021) showed that IL-7 aggravates myocardial ischemia–reperfusion injury by promoting cardiomyocyte apoptosis through its regulation of macrophage infiltration and polarization [52]. Elsewhere, Mihailovic and colleagues (2019) showed that IL-7 receptor blockade reduced post-myocardial infarction-induced atherosclerotic plaque inflammation in murine models [53]. Taken together, these findings explain the potential mechanisms by which IL-7 is involved in PAD development and progression. Second, our study unveiled a noteworthy incidence of adverse limb-related complications among patients with PAD, with nearly 10% of the cohort encountering MALEs over the span of 2 years. These findings underscore the urgent need for proactive strategies to mitigate complications in this high-risk population, emphasizing the development of more effective prognostic tools. Third, our predictive model exhibited robust performance for several reasons. Unlike conventional statistical methods like logistic regression, which assume linear correlations between independent variables and the logit of the dependent variable, advanced modeling techniques are not bound by this assumption and can adeptly capture complex non-linear relationships between inputs and outputs [54,55]. This adaptability is particularly advantageous in healthcare data, where patient outcomes are influenced by numerous factors [56]. Given the benefits of machine learning, including automation, the comprehension of non-linear relationships, and precise predictions, this technology is poised to surpass traditional statistical methods in risk prediction [54,55]. This is especially pertinent in biomarker-based models, where various proteins partake in distinct biological pathways and may interact in intricate ways to contribute to a disease process [57]. In our study, the random forest model likely achieved excellent performance due to its ensemble learning approach, which amalgamates numerous decision trees [58]. This approach not only reduces variance but also efficiently handles large datasets and minimizes overfitting [58]. In summary, our findings emphasize the advantages of employing a predictive model that integrates biomarkers, resulting in improved performance compared to relying solely on clinical information. Considering that PAD is a chronic and multifactorial condition involving diverse biological pathways, previous research has emphasized the significance of an integrated approach to enhancing PAD prognoses [59]. Our study confirms that by utilizing advanced predictive modeling techniques to analyze clinical data alongside circulating biomarkers, highly precise risk prediction tools for PAD can be developed.

### 4.4. Implications

Our predictive model based on IL-7 presents practical implications for guiding clinical decision-making across different scenarios. Initially, our tool can be employed to screen asymptomatic PAD patients, especially beneficial in family practice settings. General practitioners can incorporate the predictive model into their clinical evaluations by assessing plasma IL-7 levels and noting routine clinical features to determine a patient's PAD risk using our automated algorithm [60]. Patients identified as at high risk of PAD-related adverse events can then undergo further vascular assessment, such as arterial duplex ultrasound, to evaluate blood flow and confirm the presence of PAD [61]. Patients classified as low-risk can maintain their care under the supervision of their family physician. The focus of this care can be on optimizing risk factors through management options such as statins, ASA, and lifestyle changes [62]. On the contrary, patients identified as at high risk of MALEs should be directed to a vascular surgeon for further evaluation and management [63]. Once referred to a vascular surgeon, they can use the model in conjunction with clinical judgment to identify individuals with a high risk of developing adverse limb events who may benefit from (1) additional vascular imaging to delineate the anatomy and severity of disease [64], (2) low-dose rivaroxaban [65], and/or (3) surgical limb salvage in patients at the highest risk of adverse limb outcomes [66,67]. In summary, our automated IL-7 based tool has the potential to improve care for PAD patients in both generalist and specialist settings. It streamlines PAD screening, risk stratification, and early identification of individuals at high risk of adverse limb events. Consequently, this may decrease unnecessary specialist referrals, improve PAD outcomes, and concurrently reduce healthcare costs [68].

### 4.5. Limitations

Our study has a few limitations. First, it was conducted at a single center, necessitating further validation at other institutions to confirm the generalizability of our model. Second, the reported outcomes were based on a 2-year follow-up period, highlighting the need for longer-term observation to fully comprehend the prognostic value of our algorithm, especially given the chronic nature of PAD. Lastly, the measurement of plasma IL-7 levels is primarily utilized in research settings, underscoring the requirement for additional translational research and implementation science to demonstrate the clinical utility and feasibility of incorporating IL-7 measurement into routine care for PAD patients.

## 5. Conclusions

In this study, we identified IL-7 as a prognostic biomarker for PAD prognosis and used this circulating myokine in addition to clinical characteristics to develop a model that accurately predicted 2-year MALEs in patients with PAD. Our IL-7 myokine biomarker-based model holds promise for PAD risk stratification, offering improvements in the targeted management of PAD. Specifically, high-risk patients identified by the model can be referred for further vascular evaluation and may benefit from more aggressive medical interventions. Furthermore, our findings highlight the necessity for basic and translational studies delving into the mechanistic connections between IL-7 and the development/progression of PAD, which may strengthen our understanding of the underlying pathogenesis and inform targeted therapeutic strategies.

## Figures and Tables

**Table 1:** Baseline characteristics of individuals with and without peripheral artery disease.

	Non-PAD (*n* = 164)	PAD (*n* = 312)	*p*
Age, mean (SD)	65 (12)	71 (10)	<0.001
Female sex	67 (41)	109 (35)	0.204
Hypertension	96 (59)	257 (82)	<0.001
Dyslipidemia	100 (61)	263 (84)	<0.001
Diabetes	34 (21)	131 (42)	<0.001
Past smoking	71 (43)	171 (55)	0.001
Current smoking	35 (21)	78 (25)	0.002
Congestive heart failure	4 (2)	11 (4)	0.519
Coronary artery disease	34 (21)	118 (38)	<0.001
Previous stroke	13 (8)	51 (16)	0.011
Acetylsalicylic acid	99 (60)	251 (80)	<0.001
Statin	93 (57)	229 (73)	<0.001
ACE-Is/ARBs	74 (45)	216 (66)	0.001
Beta blockers	50 (30)	134 (41)	0.001
Calcium channel blockers	34 (21)	82 (25)	0.079
Hydrochlorothiazide or furosemide	17 (10)	41 (13)	0.190
Oral antihyperglycemic agents	8 (5)	24 (8)	0.201
Insulin	6 (4)	22 (7)	0.255

Values are reported as numbers (%) unless stated otherwise. Abbreviations: SD (standard deviation), PAD (peripheral artery disease), ARB (angiotensin II receptor blocker), ACE-I (angiotensin-converting enzyme inhibitor).

**Table 2:** Plasma concentrations of myokines in individuals with vs. without peripheral artery disease.

	Non-PAD (*n* = 164)	PAD (*n* = 312)	*p*
	Median (25%, 75% IQR)	Median (25%, 75% IQR)	
FGF-23	12.19 (9.41, 17.39)	15.33 (10.43, 23.67)	<0.001
TRAIL R2	25.65 (19.40, 35.12)	32.76 (24.37, 45.84)	<0.001
IL-7	3.41 (2.88, 4.21)	3.78 (3.14, 5.05)	0.001
MCP-1	88.27 (67.44, 115.88)	98.66 (69.15, 124.90)	0.031
LIF	10.54 (7.19, 14.40)	10.89 (7.92, 14.58)	0.246

Protein concentrations reported in pg/mL. Abbreviations: fibroblast growth factor-23 (FGF-23), interleukin-7 (IL-7), tumour necrosis factor-related apoptosis-inducing ligand receptor 2 (TRAIL-R2), leukemia inhibitory factor (LIF), monocyte chemoattractant protein-1 (MCP-1), PAD (peripheral artery disease), IQR (interquartile range).

**Table 3:** Plasma concentrations of myokines in individuals with vs. without coronary artery disease.

	No CAD (*n* = 324)	CAD (*n* = 152)	*p*
	Median (25%, 75% IQR)	Median (25%, 75% IQR)	
FGF-23	13.54 (9.46, 20.58)	15.79 (10.42, 23.43)	0.958
TRAIL R2	28.19 (20.57, 40.15)	33.38 (24.94, 46.22)	0.440
IL-7	3.63 (2.88, 4.60)	3.69 (3.16, 4.66)	0.593
MCP-1	94.00 (67.18, 123.16)	97.34 (70.18, 123.03)	0.559
LIF	10.89 (7.92, 14.19)	11.21 (8.12, 14.88)	0.876

Protein concentrations reported in pg/mL. Abbreviations: fibroblast growth factor-23 (FGF-23), interleukin-7 (IL-7), tumour necrosis factor-related apoptosis-inducing ligand receptor 2 (TRAIL-R2), leukemia inhibitory factor (LIF), monocyte chemoattractant protein-1 (MCP-1), CAD (coronary artery disease), IQR (interquartile range).

**Table 4:** Adverse events over 2 years in individuals with vs. without peripheral artery disease.

	Non-PAD (*n* = 164)	PAD (*n* = 312)	*p*
Major adverse limb event	0 (0)	28 (9)	0.001
Vascular intervention	0 (0)	19 (6)	0.001
Major amputation	0 (0)	17 (5)	0.002
Acute limb ischemia	0 (0)	0 (0)	N/A

Values are documented as numbers (%) unless stated otherwise. PAD (peripheral artery disease).

**Table 5:** Adjusted hazard ratios for associations between myokines and 2-year major adverse limb events in individuals with peripheral artery disease.

		Hazard Ratio [95% CI]	*p*-Value
IL-7	MALEs	1.56 [1.12–1.88]	0.007
	Vascular intervention	1.10 [1.05–2.98]	0.019
	Major amputation	1.02 [1.01–1.88]	0.042
TRAIL-R2	MALEs	1.00 [0.87–1.33]	0.635
	Vascular intervention	1.01 [0.99–1.43]	0.884
	Major amputation	1.01 [0.98–4.32]	0.932
MCP-1	MALEs	1.04 [0.68–1.59]	0.277
	Vascular intervention	1.11 [0.83–3.29]	0.550
	Major amputation	1.00 [0.99–1.12]	0.980
FGF-23	MALEs	1.06 [0.99–1.22]	0.484
	Vascular intervention	1.02 [0.91–1.31]	0.832
	Major amputation	1.01 [0.90–3.22]	0.742

Adjusted for sex, age, dyslipidemia, hypertension, past/current smoking, diabetes, coronary artery disease, congestive heart failure, previous stroke, statin, acetylsalicylic acid, angiotensin converting enzyme inhibitors or angiotensin II receptor blockers, calcium channel blockers, beta blockers, hydrochlorothiazide or furosemide, oral antihyperglycemic agents, and insulin. Hazard ratios were calculated for patients with PAD only, as all adverse limb events presented in patients with PAD. Abbreviations: monocyte chemoattractant protein-1 (MCP-1), tumour necrosis factor-related apoptosis-inducing ligand receptor 2 (TRAIL-R2), interleukin-7 (IL-7), fibroblast growth factor-23 (FGF-23), MALE (major adverse limb event), and CI (confidence interval).

## Data Availability

The original contributions presented in the study are included in the article; further inquiries can be directed to the corresponding author.
